# Postharvest Dehydration Temperature Modulates the Transcriptomic Programme and Flavonoid Profile of Grape Berries

**DOI:** 10.3390/foods10030687

**Published:** 2021-03-23

**Authors:** Keqin Chen, Jiahua Sun, Zhihao Li, Junxia Zhang, Ziyu Li, Li Chen, Wanping Li, Yulin Fang, Kekun Zhang

**Affiliations:** College of Enology, Viti-Viniculture Engineering Technology Center of State Forestry and Grassland Administration, Shaanxi Engineering Research Center for Viti-Viniculture, Heyang Viti-Viniculture Station, Northwest A&F University, Yangling 712100, China; chenkeqin1985@nwafu.edu.cn (K.C.); sunjiahua@nwafu.edu.cn (J.S.); lizhihao@nwafu.edu.cn (Z.L.); junxia@nwafu.edu.cn (J.Z.); lzy1999@nwafu.edu.cn (Z.L.); chenli1995@nwafu.edu.cn (L.C.); liwanping@nwafu.edu.cn (W.L.); 2019110107@nwafu.edu.cn (Y.F.)

**Keywords:** grape, dehydration, high temperature, flavonoid profile, transcriptomic programme

## Abstract

Raisins are a popular and nutritious snack that is produced through the dehydration of postharvest grape berries under high temperature (HT). However, the response of the endogenous metabolism of white grape varieties to postharvest dehydration under different temperature have not been fully elucidated to date. In this study, the white grape cultivar ‘Xiangfei’ was chosen to investigate the effect of dehydration at 50 °C, 40 °C, and 30 °C on the transcriptomic programme and metabolite profiles of grape berries. Postharvest dehydration promoted the accumulation of soluble sugar components and organic acids in berries. The content of gallic acid and its derivatives increased during the dehydration process and the temperature of 40 °C was the optimal for flavonoids and proanthocyanidins accumulation. High-temperature dehydration stress might promote the accumulation of gallic acid by increasing the expression levels of their biosynthesis related genes and regulating the production of NADP^+^ and NADPH. Compared with that at 30 °C, dehydration at 40 °C accelerated the transcription programme of 7654 genes and induced the continuous upregulation of genes related to the heat stress response and redox homeostasis in each stage. The results of this study indicate that an appropriate dehydration temperature should be selected and applied when producing polyphenols-rich raisins.

## 1. Introduction

Grapes are among the most popular fruits in the world and can not only be consumed directly but also be utilized to produce wine and raisins. With the advantages of having abundant polyphenols, a strong antioxidant capacity, a low to medium glycemic index, and a long shelf life, raisins have become a popular and healthy snack for consumers [1,2]. Meanwhile, due to the limited shelf life of table grapes, the production of raisins is also an important means of increasing the economic benefits of vineyards and avoiding waste.

Raisins are produced through the dehydration of grape berries, and the primary grape-producing countries, including China, the United States, Turkey, South Africa, Australia, and Chile, are also well-known raisin-producing countries [3]. The methods for berry dehydration can mainly be divided into natural dehydration and artificial dehydration [4]. The natural dehydration method uses natural heat and air flow to promote grape dehydration, including air drying (PAD) and sun drying (PSD) [5], while the artificial dehydration method includes external auxiliary heating and forced air flow [6]. For improved wine quality, some grapes are also dehydrated before vinification for sweet sherry wines [7]. The utilization of mild water loss technology for 3 weeks to 4 months can increase the nutrient value and volatile aroma compounds in berries [6,7,8,9,10]. During the dehydration process, the transcription profile of postharvest grape berries changed drastically. As the degree of water loss deepened, the expression levels of genes related to hormones, sugar metabolism, and defense mechanisms in the berries showed significant differences [11]. However, studies of these mild water loss techniques have largely examined the quality characteristics of grape berries with a water loss rate of only 10–40%. It has not been determined how the endogenous metabolism of grape berries changes when the water loss rate is greater than 50%. In addition, previous studies have primarily focused on forced air dehydration, but the changes in endogenous metabolism in grape berries under high temperature-induced intense dehydration have not been reported.

Among other abiotic factors, temperature has an important effect on the accumulation of secondary metabolites in grape berries during the on-vine and postharvest development stages [12,13,14,15,16,17,18]. Suitable temperatures promote the accumulation of secondary metabolites in berries, while high temperatures inhibit their biosynthesis and modulates their composition [12,13,14]. In the postharvest stage, suitable light (white light + UV light) combined with the optimal temperature (15–20 °C) can increase the expression levels of *VvCHS3*, *VvDFR*, and *VvUFGT* and promote the accumulation of anthocyanins [18]. In the process of thermal dehydration, the effect of different temperatures on the accumulation of secondary metabolites in postharvest grape berries and the involved regulatory mechanism has not been fully elucidated to date. Previous studies have primarily investigated the effects of temperature or dehydration on the biosynthesis of anthocyanins and few involved in the endogenous metabolites of white grape varieties [19]. To fully clarify the response of the endogenous metabolism of white grape varieties to different dehydration temperatures, the early maturing Chinese white grape variety ‘Xiangfei’ was investigated to determine the transcriptomic and metabolic changes undergone by postharvest grape berries. There are clear differences in the metabolites detected in grape berries subjected to different dehydration temperature modes. The results of this study indicate that intense dehydration at high temperature modulates the transcriptomic programme and flavonoid profile in postharvest grape berries.

## 2. Materials and Methods

### 2.1. Materials Collection and Experimental Layout

Grape berries of the white cultivar ‘Xiangfei’ were collected from the vineyard of Northwest A&F University in Yangling, Shaanxi Province, China. According to the commercial maturity characteristics of the variety, grape berries were harvested when the total soluble solid (TSS) content and titratable acid (TA) content of the ‘Xiangfei’ berries reached 16% and 6 g L^−1^, respectively. Fifty bunches of each variety were collected, and berries with poor maturity were removed.

A temperature-controlled incubator (Jincheng Chunlan, Jiangsu Province, HT-LRH-2 × 100, temperature setting range: 5–55 °C) was employed to perform dehydration treatment under different temperature modes. Since traditional raisins are usually produced in hot and dry regions, nine incubators were set at 30 °C (three replicates), 40 °C (three replicates), and 50 °C (three replicates), respectively, and the inner humidity was maintained at 40–50%. During the dehydration test, the water loss rate in the grape berries was recorded every 24 h. According to the characteristics of raisins, when the water loss of the berries reached 75% (and the remaining weight reached 25%), the high temperature (HT) dehydration treatment was terminated. The treated samples from each replicate were collected at the initial stage (I), the mild water loss stage (fruit water loss rate of 5%, D), the moderate water loss stage (fruit water loss rate of 50%, H), and the severe water loss stage (fruit water loss rate of 75%, Q) for quality evaluation at the molecular and metabolite levels. The berry samples at the Q stage were also named raisins in this study. Transcriptome sequencing analysis was applied to grape samples from stages I, D, H, and Q, and the metabolic profile and targeted flavonoids profile were utilized to analyze the samples from stages I and Q. However, due to the high temperature of 50 °C destroyed the nucleic acid integrity in the berries at the moderate and severe water loss stages, the samples under 50 °C at the H and Q stage were not sequenced.

### 2.2. Metabolic Profile Analysis

The grape samples freeze-dried in a vacuum were analyzed by Ultra High Performance Liquid Chromatography (UHPLC)-Electrospray ionization mass spectrometry (ESI-MS), which was performed using a Vanquish UHPLC system (Thermo Fisher, Frankfurt, Germany) coupled with an Orbitrap Q ExactiveTMHF-X mass spectrometer (Thermo Fisher, Frankfurt, Germany). A Hypersil Gold column (100 × 2.1 mm, 1.9 μm, Thermo Fisher, San Jose, CA, USA) was used to separate metabolic components from the sample. The eluents for the positive polarity mode were eluent A (0.1% formic acid in water) and eluent B (methanol), and the eluents utilized for the negative polarity mode were eluent A (5 mM ammonium acetate, pH 9.0) and eluent B (methanol). A QExactiveTMHF-X mass spectrometer was operated in positive/negative polarity mode with a capillary temperature of 320 °C, spray voltage of 3.2 kV, aux gas flow rate of 10 arb, and sheath gas flow rate of 40 arb.

Compound Discoverer 3.1 (CD3.1, Thermo Fisher, Waltham, MA, USA) was used to process the raw data files generated by UHPLC-Mass spectrometry/mass spectrometry (MS/MS) to perform peak picking, peak alignment, and quantitation for each metabolite. By comparing the retention time, *m*/*z*, and ion peak mode of the metabolites with those in the standard database and the mzCloud (https://www.mzcloud.org/, accessed on 21 August 2020) and MassBank databases (www.massbank.jp, accessed on 26 August 2020), the quality of metabolic components was determined. The peak area was used for quantitative analysis. The quality control (QC) sample was set to evaluate the system stability during the experiment, and the blank sample was used to remove background ions. The statistical software R (R version R-3.4.3), Python (Python 2.7.6 version) and CentOS (CentOS release 6.6) were used for the statistical analysis.

### 2.3. ESI-Q TRAP-MS/MS for Targeted Flavonoids Detection and Analysis

One hundred milligrams of grape samples freeze-dried in a vacuum was dissolved in 1 mL extraction solution (70% methanol/water, *v*/*v*). After the samples were vortexed at 4 °C, centrifuged (10,000× *g*, 10 min), and filtered (0.22-μm pore size), the sample extracts were analyzed using an LC-ESI-MS/MS system (HPLC, Shim-pack UFLC SHIMADZU CBM30A system, www.shimadzu.com.cn/, accessed on 21 August 2020, Kyoto, Japan; MS, Applied Biosystems 4500 Q TRAP, www.appliedbiosystems.com.cn/, accessed on 21 August 2020, Thermo Fisher, Frankfurt, Germany). Waters ACQUITY UPLC HSS T3 C18 (1.8 µm, 2.1 mm × 100 mm, Milford, MA, USA) was used as HPLC column. The water phase was ultrapure water (0.04% acetic acid added), and the organic phase was acetonitrile (0.04% acetic acid added). The analysis conditions were the following: flow rate 0.4 mL/min; column temperature 40 °C; injection volume 5 μL. The elution gradient was as follows: 100:0 *v*/*v* at 0 min, 5:95 *v*/*v* at 11.0 min, 5:95 *v*/*v* at 12.0 min, 95:5 *v*/*v* at 12.1 min, and 95:5 *v*/*v* at 15.0 min. The effluent was subsequently connected to an ESI-triple quadrupole-linear ion trap (Q TRAP)-API 4500 Q TRAP LC/MS/MS System, with which linear ion trap (LIT) and triple quadrupole (QQQ) scans were acquired. Polypropylene glycol solutions at concentration of 10 and 100 μmol/L were used for instrument tuning and mass calibration in QQQ and LIT modes, respectively. In QQQ mode, each ion pair was scanned based on the optimized de-clustering potential (DP) and collision energy (CE) [20]. Based on the local self-built database MWDB (Metware database, Metware Biotechnology Co., Ltd., Wuhan, China) of flavonoids, proanthocyanidins and other phenols, qualitative and quantitative analyses of the metabolites were performed.

### 2.4. RNA Library Construction and Sequencing

RNA extraction and integrity testing were performed according to the method of Zhang et al. [21] The RNA Nano 6000 Assay Kit of the Agilent Bioanalyzer 2100 system (Agilent Technologies, Santa Clara, CA, USA) was utilized to test the integrity of the extracted RNA. Total RNA that was determined to have acceptable integrity underwent the steps of enrichment, random interruption, reverse transcription amplification, and purification to generate double-stranded cDNA. Next, polymerase chain reaction (PCR) was performed with Phusion High-Fidelity DNA polymerase, universal PCR primers and Index (X) Primer. Finally, PCR products were purified (AMPure XP system), and library quality was assessed on the Agilent Bioanalyzer 2100 system. After clustering of the index-coded samples, the library preparations were sequenced on an Illumina NovaSeq platform, and 150-bp paired-end reads were generated.

### 2.5. Transcriptome Analysis

Clean data were acquired when the reads with adapters, N reads (N indicates that base information cannot be determined), and low-quality reads (Qphred ≤ 20 bases account for more than 50% of the total read length) were removed from the raw data. After analysis of Q20 and Q30 and GC content calculation, clean data of high quality were used for subsequent analysis.

The reference genome was downloaded from the database EnsemblPlants (http://plants.ensembl.org/info/data/ftp/index.html, accessed on 15 September 2020), and HISAT2v2.0.5 was used to construct the index of the reference genome and perform sequence alignment [22]. New genes were predicted based on StringTie (1.3.3b) [23].

Based on featureCounts (1.5.0-p3), the read numbers mapped to each gene were calculated [24]. Next, the FPKM (expected number of fragments per kilobase of transcript sequence per million base pairs sequenced) of each gene was calculated according to the length of the gene and read numbers mapped to the gene. The DESeq2 R package (1.16.1) was used to analyze the differentially expressed genes between the two comparison groups. The *p* value was adjusted to control the false discovery rate based on the method of Benjamini and Hochberg. Genes with corrected *p* values < 0.05 were classified as DEGs.

The Kyoto Encyclopedia of Genes and Genomes (KEGG, http://www.genome.jp/kegg/, accessed on 15 September 2020) is a useful database resource for analyzing genome sequencing data and gene functions. The integrated database resource consists of eighteen databases, which are broadly categorized into systems information, genomic information, chemical information and health information. ClusterProfiler (3.4.4) was also used to analyze the enrichment of DEGs in KEGG pathways.

### 2.6. qRT-PCR Analysis

Total RNA isolation, cDNA synthesis and gene expression analysis were performed according to our previously described method [25]. Primer Premier 6.0 was used for primer design (Table S1), and *ubiquitin* and *EF1γ* were used as internal standards for expression normalization. RT-qPCR was performed using the CFX96 Real-Time PCR Detection system (Bio-Rad, Hercules, CA, USA), and gene expression levels were calculated based on the 2^−△△Ct^ method.

### 2.7. Determination of Sugar Components and Organic Acid Components

HPLC (Agilent, USA) was used to detect soluble sugars and organic acid components based on the modified method of Liu et al. [26] The Agilent Zorbax Carbohydrate column was used to separate the sugar component, and the flow rate of the mobile phase (75% acetonitrile: 25% water, *v*/*v*) was set as 1 mL/min, the coupled detector was the G1362A refractive index detector (RID, Agilent, USA), and the analysis conditions were the following: column temperature 35 °C; injection volume 10 μL. The Agilent SB-AQ column was used to separate the organic acid components, and the mobile phase (5% methanol: 95% 10 mM KH_2_PO_4_ solution, *v*/*v*, pH = 2.6) flow rate was set to 0.8 mL/min, the coupled detector was the G1314B variable wavelength ultraviolet detector (VWD, Agilent, USA), and the analysis conditions were the following: column temperature 25 °C; injection volume 10 μL. Standards of glucose, fructose, sucrose, malic acid, tartaric acid, shikimic acid, lactic acid, succinic acid, citric acid, and acetic acid (ShanghaiyuanyeBio-Technology Co., Ltd., Shanghai, China) were used for qualitative and quantitative analysis of the samples.

### 2.8. Determination of NADP^+^ and NADPH Content

A NADP^+^/NADPH detection kit (WST-8 method, Beyotime, Shanghai, China) was used to determine the NADP^+^ and NADPH contents. According to the instruction manual, NADP^+^/NADPH extract was employed to homogenize the ground sample. After centrifugation, the absorbance of the homogenate with addition of G6PDH chromogenic solution was detected under 450-nm ultraviolet light to determine the total content of NADP^+^ and NADPH (NADP^+^_total_). Subsequently, the homogenate was heated at 60 °C to decompose NADP^+^, and the absorbance was detected again for the absorbance of NADPH. The content of NADP^+^_total_ and NADPH was calculated according to the NADPH standard curve by absorbance and the content of NADP^+^ was calculated by NADP^+^_total_ content minus NADPH content.

### 2.9. Statistical Analysis

Statistical analysis was performed with the software SPSS Statistics 22 (IBM, Armonk, NY, USA). Differences among samples under different dehydration stages and temperatures were analyzed by a two-way ANOVA followed by the Duncan’s multiple comparison test at *p* < 0.05. Differences among samples at the same dehydration stage but under different temperatures were analyzed by a one-way ANOVA followed by the Duncan’s multiple comparison test at *p* < 0.05. The normalized transcriptome data after taking the logarithm (LogFPKM) and the metabolites content data were used for principal component analysis (PCA), respectively. The normalized metabolites content data and the targeted flavonoids content data after taking the logarithm was prepared for making the heatmap by the Toolbox for Biologists (TBtools, v1.082, Guangzhou, China). The pie chart and line chart were presented through OriginPro 9.1 (OriginLab Corporation, Northampton, MA, USA).

## 3. Results

### 3.1. Changes in Water Content, Soluble Sugar Components, and Organic Acid Components in Grape Berries Subjected to HT Dehydration Mode

**Figure 1:**
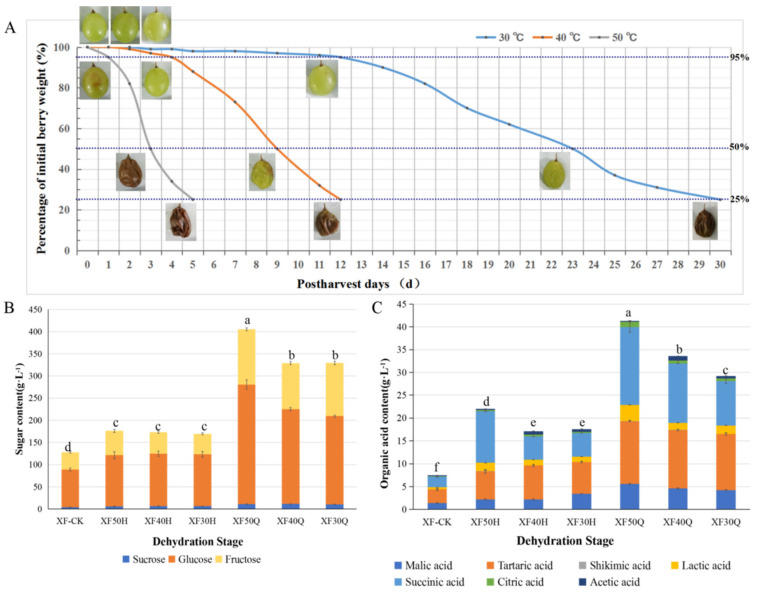
Effect of dehydration temperature on berry physiological indexes on cv. Xiangfei. (**A**). Effect of different dehydration temperature on the berry morphology and water loss rate. (**B**). Content of sugar components in berries at different dehydration stage. Error bars represent the standard deviation between three replicates, the letters in the column chart represent the significance of the total soluble sugar content differences among samples at *p* < 0.05 (Duncan’s multiple comparison test). The same is true below. XF-CK represent grape berries at the initial stage. XF50H, XF40H, XF30H represent samples at the moderate dehydration stage under 50 °C, 40 °C, and 30 °C, respectively. XF50Q, XF40Q, XF30Q represent samples at the severe dehydration stage under 50 °C, 40 °C, and 30 °C, respectively, the same below. (**C**). Content of organic acid components in berries at different dehydration stage. The letters in the column chart represent the significance of the total acid content differences among samples at *p* < 0.05 (Duncan’s multiple comparison test).

Postharvest grape berries underwent dehydration treatments with different heating temperatures, and their morphological changes during the water loss process were measured (Figure 1A). The water loss rate of the berries was the highest at 50 °C, and the weight loss rate reached 75% after 5 days of treatment, while the water loss rate was the lowest at 30 °C, with the weight loss rate reaching 75% after 30 days of treatment. Meanwhile, the different HT dehydration treatments were also distinguished by changes in berry morphology. The berry appeared brown when the water loss rate reached 5% at 50 °C, 50% at 40 °C, or 75% at 30 °C, indicating that the dehydration temperature not only changed the water loss rate but also changed the content of endogenous metabolites in the berries.

Compared with the berries in the initial stage of dehydration (XF-CK), the contents of total soluble sugar and organic acid increased in both the severe and moderate water loss stages (Figure 1B,C), which may be observed partly because water loss induced an increase in metabolite content per unit weight. However, notably, the sugar and acid contents did not increase in proportion to the decrease in grape weight, indicating that some sugar and acid components were also transformed during the process of dehydration. The changes in the content of sugar and organic acid components of grape berries also exhibited differences under different temperatures. In the severe water loss stage (75%), the contents of glucose, fructose and total soluble sugars and the contents of malic acid, tartaric acid, lactic acid and citric acid in grape berries treated with 50 °C were greater than those of other treatments, while the content of acetic acid in berries treated with 40 °C and 30 °C was higher. Different temperature conditions, especially at 40 °C and 30 °C, may activate a variety of sugar and acid metabolism pathways in berries.

### 3.2. Differences in the Metabolic Profiles of Raisins Produced under Different HT Dehydration Modes

**Figure 2:**
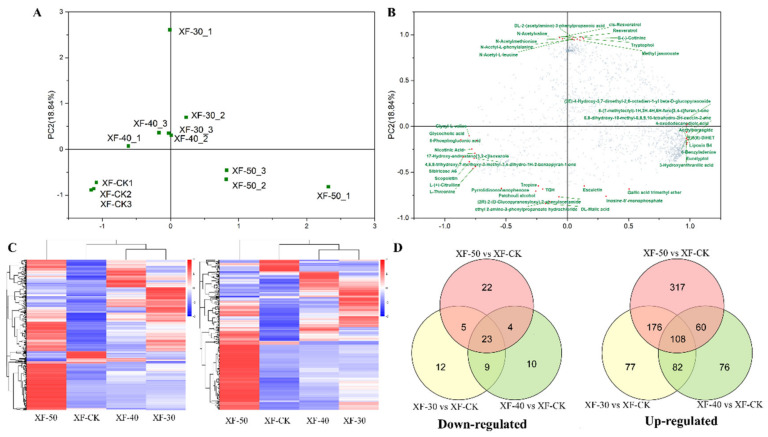
Overview of metabolic profiles in raisins produced at different dehydration temperature. (**A**) Principal component analysis (PCA) scatter plot of different raisins based on the metabolic profiles. The metabolites content data were used for the analysis. XF-CK represent grape berries at the initial stage. XF-30, XF-40, XF-50 represent ‘Xiangfei’ raisins (samples at the Q stage) produced under 30 °C, 40 °C, and 50 °C, respectively, and the number 1, 2, 3 behind them represent the three replicate samples. The same below. (**B**) PCA loading plot of metabolic components. The 10 components with the highest and lowest scores in PC1 and PC2 were marked. (**C**) Cluster heat map of metabolic profiles of different raisins. The normalized metabolites content data after taking the logarithm was prepared for making the heatmap. The first map represent metabolic profiles detected in positive mode, while the second one represent those detected in negative mode. (**D**) Venn diagram of the number of downregulated and upregulated components in different comparison group. Sample1 vs. Sample2 represent the number of differential metabolites in the former (Sample1) compared with the latter (Sample2).

Through metabolic profile analysis, 1760 components (1128 in positive mode and 632 in negative mode) were identified in the ‘Xiangfei’ berries. Principal component analysis showed the differences in metabolites between the samples (Figure 2A). The scatter plot showed that there were considerable differences between the raisin samples or between the raisins and the berries. The scattered points of grape berries at the initial stage (XF-CK) were mainly distributed in the negative semi-axis areas of PC1 and PC2, while those of raisin samples at the Q stage (XF-30, XF-40, and XF-50) were mainly distributed in other quadrants. Except for XF-30-1, the scattered spots of raisins dehydrated at 30 °C and 40 °C were clustered together, indicating that their metabolites were similar (Figure 2A). The metabolites detected in the positive and negative modes were analyzed by cluster heat map, and a similar result was also obtained (Figure 2C). In the load diagram, the 10 components with the highest and lowest scores in PC1 and PC2 were marked. Sugars, acids, polyphenols, and esters and other substances were included, which indicated that various primary and secondary metabolic pathways were functioning in the process of grape dehydration (Figure 2B). Meanwhile, it could be observed from the cluster heat map that the content of most metabolites in XF-CK was relatively low, while there were specific metabolic components with relatively high content in XF50, XF40, and XF30 samples. The content of metabolites in grape berries was mostly lower than that in raisins (Table S2), and the increase in the content of some components was considerably greater than the decrease in berry weight. The content of cis-resveratrol increased sixfold in raisins at 30 °C but only changed insignificantly in raisins treated at other temperatures, indicating that the operating efficiency of some metabolic pathways during dehydration was also temperature-dependent.

The Venn diagram reflected the number of specific and common regulated substances in each comparison group (Figure 2D). Compared with grape berries in the initial stage, at 30, 40, and 50 °C, there were 49, 46, and 54 downregulated metabolites in dehydrated raisins, respectively, and there were 443, 327, and 661 upregulated metabolites, respectively. The number of upregulated and downregulated differential metabolites in samples treated with 50 °C was the largest. In an analysis of the comparison groups, it was found that there were 23 common downregulated components and 108 common upregulated components in ‘Xiangfei’ raisins. The above analysis indicated that there was a specific correlation between metabolite accumulation and different dehydration temperatures. 

From the perspective of the enrichment pathway of differential metabolites (Figure S1), compared with XF-CK, the enrichment pathways of differential metabolites shared by different HT dehydration modes are concentrated in 16 metabolic pathways, including alpha-linolenic acid metabolism, tryptophan metabolism, arginine biosynthesis, aminoacyl-tRNA biosynthesis, monoterpenoid biosynthesis, vitamin B6 metabolism, zeatin biosynthesis, phenylpropanoid biosynthesis, indole alkaloid biosynthesis, glycine, serine and threonine metabolism, riboflavin metabolism, tyrosine metabolism, purine metabolism, metabolic pathways, galactose metabolism, and glucosinolate biosynthesis. Five, six, and twelve pathways were specifically enriched in the comparison groups of XF-30 vs. XF-CK, XF-40 vs. XF-CK, and XF-50 vs. XF-CK, respectively. Some metabolic pathways related to nutritional quality, such as stilbenoid, diarylheptanoid, and gingerol biosynthesis, were specifically enriched in the XF-30 vs. XF-CK comparison group, flavone and flavonol biosynthesis in the XF-40 vs. XF-CK group, and biosynthesis of unsaturated fatty acids in the XF-50 vs. XF-CK group.

From the comparison of raisins, 6, 3, and 9 metabolic pathways were enriched in the comparison groups of XF-50 vs. XF-30, XF-40 vs. XF-30, and XF-50 vs. XF-40, respectively (Figure S2). There were seven common differential metabolite enrichment pathways between the comparison groups, including plant hormone signal transduction, flavonoid biosynthesis, phenylpropanoid biosynthesis, ATP-binding cassette (ABC) transporters, metabolic pathways, flavone and flavonol biosynthesis, and tyrosine metabolism. In these pathways, the flavonoid biosynthesis and flavone and flavonol biosynthesis pathways were closely related to the nutritional value of raisins and were also related to consumers’ preferences. Therefore, we performed targeted detection of flavonoids in the samples.

### 3.3. Difference in Flavonoids, Proanthocyanidins and Other Phenols in Raisins Produced under Different HT Dehydration Modes

**Figure 3:**
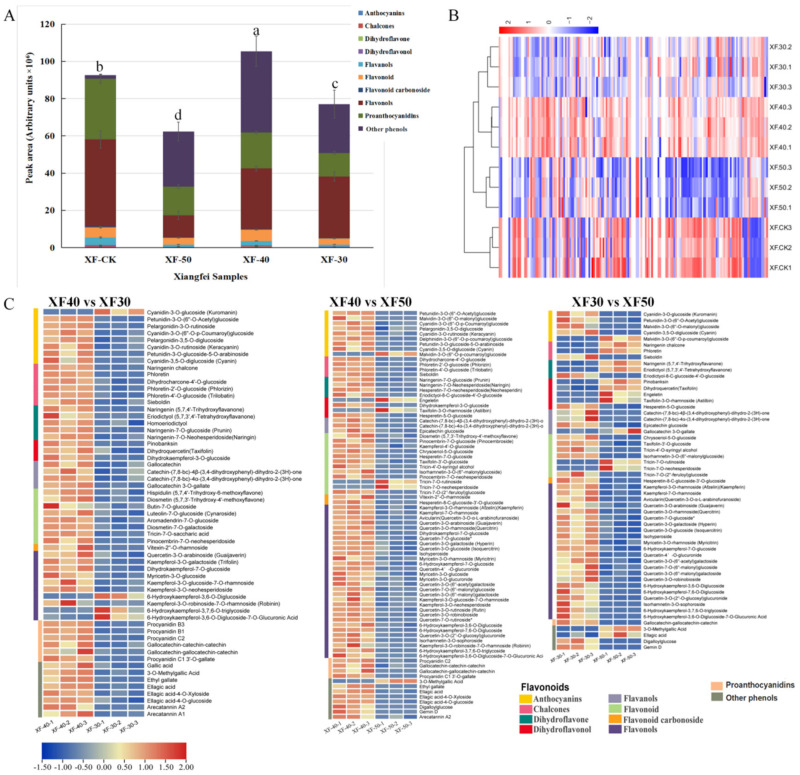
Overview of targeted flavonoids in ‘Xiangfei’ raisins produced at different dehydration temperature. (**A**)**.** Peak area of different subclass of flavonoids, proanthocyanidins and other phenols in raisins produced under different dehydration temperature. Error bars represent the standard deviation between three replicates, the letters in the column chart represent the significance of the total content differences among samples at *p* < 0.05 (Duncan’s multiple comparison test). (**B**). Cluster heat map of phenolic components in different raisins. The normalized phenolic components content data after taking the logarithm was prepared for making the heatmap. The more intense the red, the higher the content, and the more intense the blue, the lower the content. The same below. (**C**). Heat map of differential flavonoids, proanthocyanidins and other phenols in raisins produced under different dehydration temperature.

A total of 146 flavonoids were identified in this study (Table S3), including 21 anthocyanins, 6 chalcones, 7 dihydroflavones, 8 dihydroflavonols, 10 flavanols, 41 flavonoid, 5 flavonoid carbonosides, and 48 flavonols. A total of 10 proanthocyanidins and 15 other phenols (mainly gallic acid and its derivatives) were also identified. The content of these identified phenolic components in raisins and grape berries were notably different (Figure 3A). Proanthocyanidins and flavonols accounted for a larger proportion in ‘Xiangfei’ grapes, while proanthocyanidins, flavonols, and gallic acid and its derivatives were the main components in raisins. Except for the subgroup of other phenols, the content of various flavonoids and proanthocyanidins in raisins was lower than that in grape berries at the initial stage. From the comparison between the raisins, the contents of various flavonoids in the raisins produced under the dehydration treatment at 40 °C were generally greater than those in the samples produced at 30 °C and 50 °C. Compared with grape berries, the component with the most significant increase in the content of raisins produced at 40 °C was gallic acid and its derivatives. The cluster heat map showed that the dehydration of grape berries modulated the accumulation of flavonoids proanthocyanidins and other phenols (Figure 3B). In general, the raisin samples were clustered into one category, and the distance between the raisins and the grapes was relatively large. The comparison between the raisin samples showed that the samples from 30 °C and 40 °C dehydration were clustered together, indicating that these identified phenolic components in these samples were similar and were clearly different from those in samples produced at 50 °C.

The Venn diagram shows the specific or common changes in these identified phenolic components contents in grape berries under different HT dehydration modes (Figure S3A). Compared with the initial grape berries (XF-CK), 25 flavonoids in raisins were commonly downregulated, and 19 components were upregulated. Compared with XF-CK, raisins from 50 °C dehydration have the most specific downregulated substances, while raisins from 40 °C have almost no specific downregulated components. At the same time, compared with CK, the 40 °C dehydration treatment also specifically induced increases in the content of seven components. Combined with the results of volcano map (Figure S3B,C), it could be concluded that the accumulation of flavonoids proanthocyanidins and other phenols in grapes during dehydration is affected by temperature. The contents of most these identified phenolic components showed a downward trend at 50 °C, while some flavonoids accumulated at 40 °C and 30 °C (Figure S4).

The differential flavonoids, proanthocyanidins and other phenols observed in raisins produced under different HT dehydration modes are listed (Figure 3C). Fifty-nine, 80, and 55 phenolic components were identified as differential metabolites in the comparison groups of XF40 vs. XF30, XF40 vs. XF50, and XF30 vs. XF50, respectively. Compared with XF40, almost all (55/59, 74/80) flavonoids, proanthocyanidins, and other phenols in XF30 and XF50 were reduced in content. Compared with XF30, the content of 14 components was higher, and the content of 41 components was lower, in XF50. The above results indicated that compared with the 30 °C and 50 °C dehydration modes, the 40 °C dehydration mode was more conducive to the accumulation of most of the flavonoids, proanthocyanidins, and other phenols in raisins.

The top 10 identified phenolic components with the most significant increases and decreases in different comparison groups are listed (Figure S5). Compared with the berries in the initial stage, ethyl gallate (pme0310) was the component with the most notable increase in content in the raisins produced under each dehydration mode. The components with the most reduced content in raisins were petunidin-3-*O*-glucoside-5-*O*-arabinoside (Smsp001640) at 30 °C and kuromanin (pmb0550) at 40 °C or 50 °C. Compared with XF40, sieboldin (Hmpn005101), and procyanidin C1 3′-O-gallate (Zmmp003562) were the most downregulated components in XF50 and XF30, respectively, while ethyl gallate (pme0310) and kuromanin (pmb0550) were the most upregulated components. Based on the analysis described above, the HT dehydration mode hindered the accumulation of proanthocyanidins, flavonols, flavanols, and other flavonoids in postharvest grape berries but promoted the biosynthesis of gallic acid and its derivatives. The temperature of 40 °C was the best for the accumulation of phenolic components in the ‘Xiangfei’ berries during the HT dehydration process.

### 3.4. Transcriptome Analysis in Grape Berries under Different HT Dehydration Modes

We extracted RNA from postharvest grape berries under various dehydration temperature modes (30 °C, 40 °C, and 50 °C) at the initial stage, mild water loss (5%) stage, moderate water loss (50%) stage and severe water loss (75%) stage. Complete RNA could not be obtained from grape berries at the moderate and severe water loss stages at 50 °C, and only one replicate of samples at the severe water loss stage at 30 °C could provide useful RNA. Complete RNA from eight groups, including XF-CK (grape berries at stage I), XF30 (grape berries at stage D, H, and Q), XF40 (grape berries at stage D, H, and Q), and XF50 (grape berries at stage D) was eventually used for sequencing and comparative analysis. The average number of clean reads obtained after quality control of the sequencing data was 43,574,155, and the average mapping rate to the grape genome data (http://plants.ensembl.org/info/data/ftp/index.html, accessed on 21 August 2020) was 88% (Table S4).

**Figure 4:**
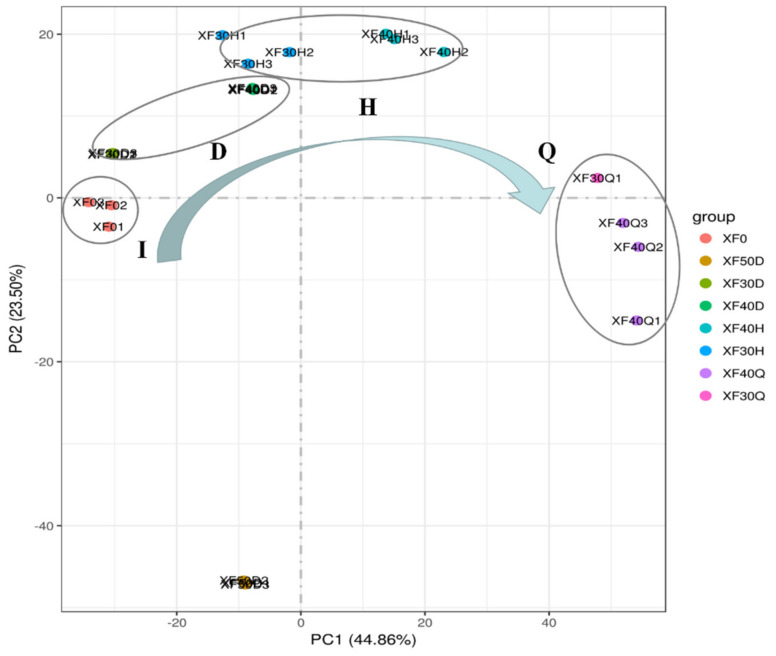
PCA scatter plot of samples dehydrated under different temperature based on their transcriptome data. The normalized transcriptome data after taking the logarithm were used for PCA. The samples in the same dehydration stage were wrapped by black solid circles. I, D, H, and Q represent the initial berry stage, the mild dehydration stage, the moderate dehydration stage and the severe dehydration stage, respectively. XF0 represent the samples at the initial berry stage.

From the distribution of scattered points, the score of each group of samples under 30 °C and 40 °C dehydration modes showed regular changes (Figure 4). With the increase in the water loss rate, the distance between the berry samples and CK became greater, especially in the direction of PC1. The scattered points of samples at the initial stage were mainly distributed at the top of the negative semiaxis of PC1, and their position gradually moved to the positive semiaxis as the water loss rate increased. Meanwhile, the PC1 score of the sample treated at 40 °C was greater than that of the sample treated at 30 °C under the same dehydration state, which indicated that PC1 was closely related to the dehydration state of grape berries and the temperature change from 30 °C to 40 °C.

In the direction of the PC2 axis, the distance between the grapes treated at 50 °C and other samples was relatively long. The PC2 axis might be related to the combined effects of high temperature and other factors (Figure 4). Based on the scores of PC1 and PC2, the gene transcription programme in grape berries treated with 30 °C and 40 °C could be rhythmically regulated partly by temperature and water loss rate, while the gene transcription in grape berries treated with 50 °C was notably different from that of other samples.

### 3.5. Changes in the Transcriptome Programme in Grape Berries under Different Dehydration States

Previous studies have shown that the use of forced air to accelerate water loss could promote transcriptomic programme development in postharvest grape berries [27]. To determine the effect of temperature on gene transcription, the transcriptomic programme of grape berries under different HT dehydration states was also studied. Because a high temperature of 50 °C destroyed the nucleic acid integrity in the berries at the moderate and severe water loss stages, only the transcription patterns in grape berries at various water loss stages at 30 °C and 40 °C were analyzed. A temperature of 30 °C was used as a control condition for thermal dehydration. Compared with 30 °C, the dehydration rate of berries under 40 °C was more rapid, and the transcription programme of some genes (7654) during the dehydration development stages (I to Q stage) was accelerated.

**Figure 5:**
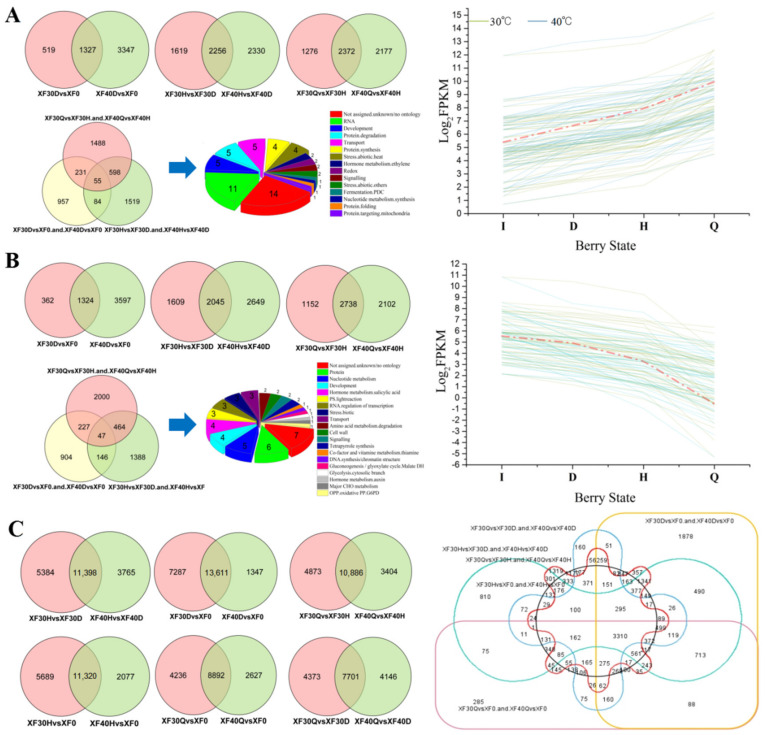
Comparison of differentially expressed genes (DEGs) in samples treated at 30 °C and 40 °C in different dehydration stages. (**A**). Screening for up-regulated genes between different dehydration stage in samples treated at 30 °C and 40 °C. Venn diagram showed the common up-regulated differential genes in comparison groups at different dehydration stage. Pie diagram showed the function annotation of common up-regulated differential genes in samples treated with 30 °C and 40 °C from the initial stage to severe dehydration stage. Line chart showed transcription program of common up-regulated differential genes in samples treated with 30 °C and 40 °C. (**B**). Screening for down-regulated differential genes between different dehydration stage in samples treated at 30 °C and 40 °C. Venn diagram, Pie diagram, and Line chart was also used to show the characteristic of down-regulated differential genes. (**C**). Screening for genes with no significant differences in expression between different dehydration stages in samples treated at 30 °C and 40 °C. The 2-element Venn diagram and the 6-element Venn diagram were used to represent the number of genes with no difference in expression between different groups.

Among differentially expressed genes (DEGs), all the upregulated at each dehydration developmental stage (Figure 5A) were screened out. The Venn diagram shows the overlap of upregulated genes in the berries treated with 30 °C and 40 °C in different dehydration states. Compared with the initial state, 1327 genes were commonly upregulated in the mild water loss (5%) stage at 30 °C and 40 °C. Compared with the mild water loss state, a total of 2256 genes were commonly upregulated in the moderate water loss state. Compared with the moderate water loss state, 2372 genes were commonly upregulated in the severe water loss state (water loss rate 75%). After the integrated analysis of the genes upregulated at each stage, 55 genes were screened out and determined to be continuously upregulated during the process of berry dehydration. When function annotated by MapMan, eleven of these continuously upregulated genes were found to be involved in the RNA transcription regulation process, including MYB, bHLH, and bZIP transcription factor family members (Table S5). Five genes were annotated to be involved in protein degradation related to ubiquitin, and four genes were related to heat stress. Two genes were related to the induction and signal transduction of ethylene, and two genes were related to redox homeostasis. Dehydration might activate multiple metabolic pathways in the berries, in which ethylene signal transduction, protein ubiquitination degradation, and redox homeostasis are involved, and various regulatory factors might participate in the signal transduction process.

The genes that were downregulated in the berries at each dehydration development stage at 30 °C and 40 °C were also screened, and the specific distribution of genes in each comparative group is shown by a Venn diagram (Figure 5B). Compared with the initial state, 1324 genes were commonly downregulated in the mild water loss (5%) stage at 30 °C and 40 °C. Compared with the mild water loss state, a total of 2045 genes were commonly downregulated in the moderate water loss state. Compared with the moderate water loss state, 2738 genes were commonly downregulated in the severe water loss state (water loss rate 75%). Through integration analysis, 47 genes that were continuously downregulated during the berry dehydration process were screened out. Among these genes, four were involved in the biosynthesis and degradation of salicylic acid, three were involved in light reactions, three belonged to the Aux/IAA family, C3H zinc finger family, and WRKY domain transcription factor family, and six were involved in protein degradation and posttranslational modification.

Through a comprehensive comparison of samples in the four dehydration stages, genes with no significant transcription differences among them were also screened out (Figure 5C). Regardless of the treatment was performed at 30 °C or 40 °C, the expression levels of 3310 genes were not significantly different between each dehydration stage, the function of which was involved in such processes as amino acid metabolism, hormone metabolism, lipid metabolism, and posttranslational protein modification. Some stages of these processes or pathways might not be affected by dehydration and temperature.

**Figure 6:**
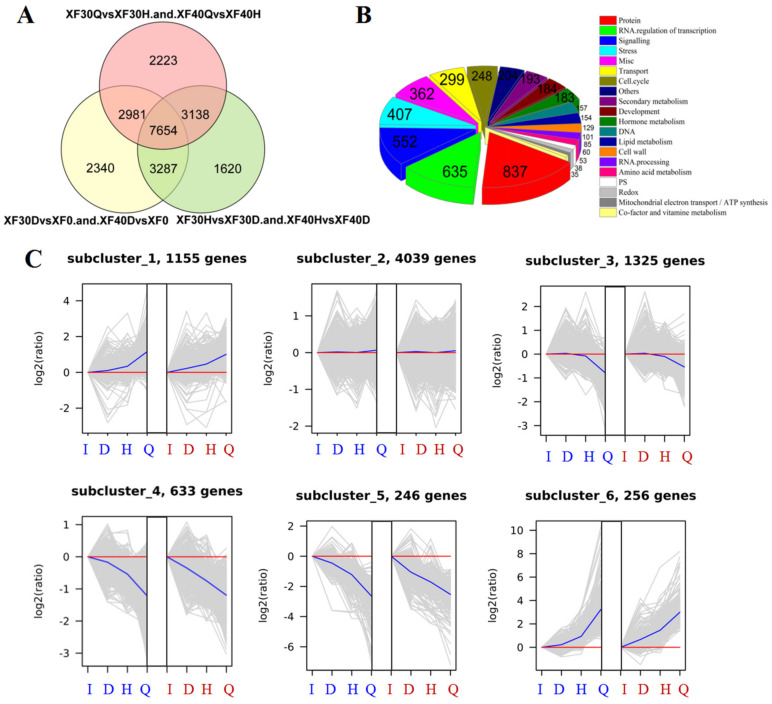
Characteristics of the genes with similar transcription program between samples treated at 30 °C and 40 °C during the whole dehydration process. (**A**). Screening for genes with similar transcription program between samples treated with 30 °C and 40 °C. (**B**). Pie diagram showed the function annotation of the ‘consistent’ genes. (**C**). Transcription program of the ‘consistent’ genes. The abscissa of the line chart was the sample name. The blue “I, D, H, Q” indicated the dehydration stages under 30 °C, while the red one indicated that under 40 °C. The ordinate was the expression value after logarithmic centering correction. The gray line in each sub-graph represents the gene expression level of a cluster after relative correction under different experimental conditions. The blue line represents the average of the gene expression level of all genes in this cluster after relative correction.

Through the integration of the above mentioned specific and common genes during the dehydration process, those with consistent transcription programs between 30 °C and 40 °C were screened out. From the initial stage to the mild dehydration stage, a total of 16,262 genes showed the same change pattern (including upregulation, downregulation and no significant difference) between the 30 °C and 40 °C modes. From the mild to moderate dehydration stage, a total of 15,699 genes showed the same pattern, and from moderate dehydration to severe dehydration, 15,996 genes showed the same change pattern between the 30 °C and 40 °C modes. Considering the four dehydration stages, 7654 “consistent” genes maintained their transcription programme in berries even if the dehydration temperature mode changed from 30 °C to 40 °C (Figure 6A). The functions of these consistent genes were related to proteins, transport, RNA, and cells, and the metabolic pathways involved included secondary metabolism, hormone metabolism, lipid metabolism, and amino acid metabolism (Figure 6B). These consistent genes were clustered through the H-cluster algorithm when their expression was centrally corrected, and their transcription programs at 30 °C and 40 °C are depicted by cluster line graphs (Figure 6C). Differential genes were divided into six subclusters, and genes in the same subcluster had similar transcription patterns during dehydration. The gene expression of subclusters 1 and 6 showed a gradually increasing pattern, subclusters 3, 4, and 5 showed an overall decreasing pattern, and the expression level of subcluster 2 generally changed only slightly.

To accurately evaluate the stability of key metabolic pathways between the 30 °C and 40 °C modes, the ratio of the number of consistent genes annotated in the pathway to the total number of genes annotated in the pathway by MapMan was calculated (Table S5). Only 30% of genes were involved in amino acid metabolism, 20% in hormone metabolism, 26% in lipid metabolism, 24% in regulation of transcription, 15% in flavonoid metabolism, 13% in isoprenoid metabolism, 20% in lignin biosynthesis, and 21% in stress response showed a constant transcriptomic programme between 30 °C and 40 °C. In the shikimate biosynthesis pathway, 5 (VIT_00s0391g00070; VIT_14s0030g00660; VIT_14s0068g01460, VIT_06s0004g02960) of the 18 identified genes belonged to the consistent category, indicating that there were differences in the transcriptional level of the shikimate biosynthesis pathway between 30 °C and 40 °C. The temperature of 40 °C not only accelerated the change in the water loss rate in the berries but also changed the evolution pattern of a large number of genes during dehydration.

Compared with the dehydration treatment at 30 °C, the accelerated dehydration treatment at 40 °C could also accelerate the transcription process of some genes without changing their expression pattern. The expression pattern of these genes was not sensitive to changes in dehydration temperature (30, and 40 °C) but tended to change depending on the dehydration state or water loss ratio.

### 3.6. Analysis of Differentially Expressed Genes under Different Dehydration Temperatures

**Figure 7:**
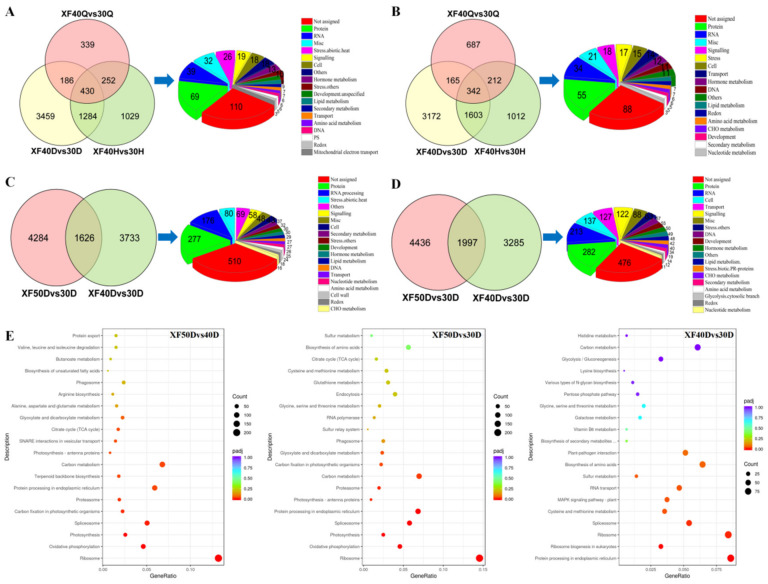
Comparison of DEGs in samples treated at different dehydration temperature. (**A**). The up-regulated genes in samples under 40 °C compared with those under 30 °C. Pie diagram showed the function annotation of those commonly regulated genes during the whole dehydration process. The same below. (**B**). The down-regulated genes in samples under 40 °C compared with those under 30 °C. (**C**). The up-regulated genes in samples under 50 °C compared with those under 30 °C and 40 °C at the mild dehydration stage. Pie diagram showed the function annotation of those commonly regulated genes in the mild dehydration stage. The same below. (**D**). The down-regulated genes in samples under 50 °C compared with those under 30 °C and 40 °C at the mild dehydration stage. (**E**). The Kyoto Encyclopedia of Genes and Genomes (KEGG) pathways enriched with up-regulated DEGs between different groups. GeneRatio represents the ratio of the number of differential genes annotated to the KEGG pathway to the total number of differential genes. Count represents the number of differential genes annotated to the KEGG pathway. padj represents the *p*-value corrected by multiple hypothesis testing.

Compared with the 30 °C dehydration mode, the number of genes upregulated at each dehydration stage at 40 °C is shown in Figure 7A, and the number of downregulated genes is shown in Figure 7B. The expression levels of 430 genes were upregulated, and 342 genes were downregulated, in each developmental stage. In addition to the genes involved in the synthesis, development, and degradation of proteins and RNA, the number of genes involved in the heat stress response and signal transduction was the largest among the upregulated genes, which indicated that the heat shock response in the berries under 40 °C was more pronounced. Meanwhile, the expression of genes related to redox and mitochondrial electron transport was also activated at 40 °C, and they might be involved in the stabilization of the redox state under heat stress.

In the mild dehydration stage, the differentially expressed genes between the dehydration temperatures of 50 °C, 40 °C, and 30 °C were compared. Compared with the 30 °C treatment, 1626 genes were commonly upregulated at 50 °C and 40 °C (Figure 7C), while 1997 genes were commonly downregulated (Figure 7D). Among the upregulated genes, except for those related to proteins, RNA, and miscellaneous gene products, the number of genes enriched in heat stress response and signal transduction pathways was the largest, indicating that heat stress-related genes were all active regardless of long-term heat stress or short-term heat treatment. In addition, some of the upregulated genes and downregulated genes were involved in the same metabolic process and signal transduction pathway, reflecting the complexity of the transcription-level response of grape berries to high temperature and dehydration stress.

Compared with the 30 °C and 40 °C treatments, the differentially upregulated genes in 50 °C were significantly enriched in ribosomes, oxidative phosphorylation, photosynthesis, spliceosomes, protein processing in the endoplasmic reticulum, carbon metabolism, proteasomes, and glyoxylate and dicarboxylate metabolism (Figure 7E). Compared with the 30 °C treatment, the upregulated differentially expressed genes at 40 °C were significantly enriched in protein processing in the endoplasmic reticulum, ribosome biogenesis in eukaryotes, ribosomes, spliceosomes and the mitogen-activated protein kinase (MAPK) signalling pathway. Compared with the 30 °C and 40 °C treatments, the downregulated differentially expressed genes at 50 °C were significantly enriched in the mRNA surveillance pathway, the MAPK signalling pathway, and RNA degradation (Figure S6). Ribosomes are important complexes for RNA translation. It could be observed from the metabolic pathways enriched in the high-temperature dehydration mode that high-temperature stress affected the transcription, splicing, and translation process of RNA.

Compared with the 30 °C treatment, a higher dehydration temperature stimulated a variety of signal transduction pathways related to the heat shock response and redox homeostasis. The specific and common signal transduction pathways and transcription factors related to dehydration and temperature might partly explain the differential accumulation of metabolites under different HT dehydration modes.

### 3.7. Differences in the Gallic acid Biosynthesis Pathway in Grape Berries under Different HT Modes

**Figure 8:**
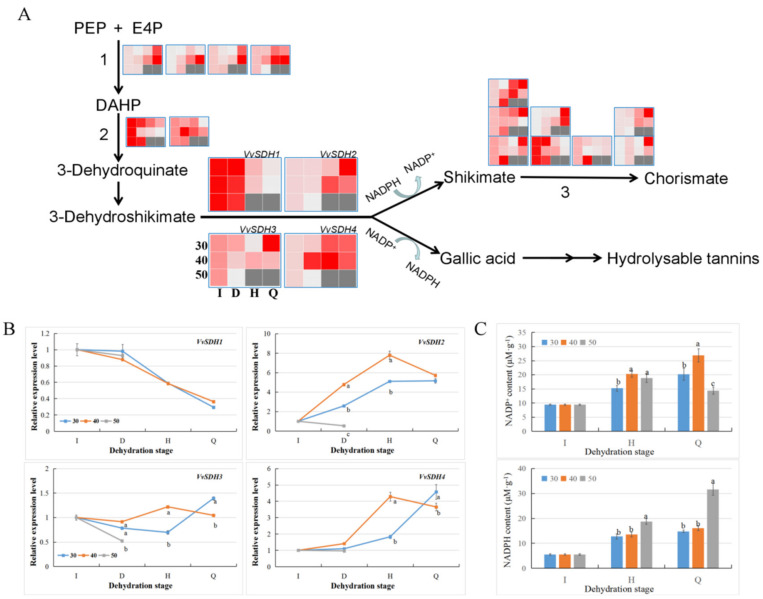
Changes in the shikimic acid pathway of berries during high temperature (HT) dehydration. (**A**). The expression levels of genes in shikimic acid pathway. 1 represents 3-deoxy-D-arabino-heptulosonate 7-phosphate synthase and the four heat maps represent the transcription pattern of its four members (VIT_00s0391g00070, VIT_00s1217g00010, VIT_18s0001g06250, VIT_00s0591g00020), respectively. 2 represents 3-dehydroquinate synthase (VIT_04s0023g03820, VIT_14s0108g00350). 3 includes shikimate kinase (VIT_14s0068g01460, VIT_18s0001g01730, VIT_07s0031g01600, VIT_16s0039g02500. VIT_02s0012g01800) and 5-enolpyruvylshikimate-3-phosphate synthase (VIT_15s0048g00350), chorismate synthase (VIT_06s0004g02960, VIT_13s0019g04190). The normalized transcriptome data of these specific genes after taking the logarithm (LogFPKM) was prepared for making the heatmap. The more intense the red, the higher the expression level. (**B**). The expression levels of *VvSDHs* based on RT-qPCR. Error bars represent the standard deviation between three replicates, the letters in the line chart represent the significance of the differences among samples at the same dehydration stage at *p* < 0.05 (Duncan’s multiple comparison test). (**C**). Content of NADP^+^ and NADPH in berries at different dehydration stage. Error bars represent the standard deviation between three replicates, the letters in the column chart represent the significance of the differences among samples at the same dehydration stage at *p* < 0.05 (Duncan’s multiple comparison test).

As the contents of other phenols, such as gallic acid (GA) and ellagic acid, increased significantly under the HT dehydration mode, we analyzed the gallic acid biosynthesis-related shikimic acid (SA) metabolic pathways based on the transcriptomic data (Figure 8). During the dehydration process, the gene expression of 3-deoxy-D-arabino-heptulosonate 7-phosphate synthase in berries at 30 °C and 40 °C showed a gradual increasing trend, especially at 40 °C, under which the expression level of its members was higher than that at 30 °C (Figure 8A). However, the expression levels of the 3-dehydroquinate synthase gene exhibited differences. From the mild water loss stage, the expression level of VIT_04s0023g03820 in berries at 30 °C was higher than that in berries at 40 °C, while the expression level of VIT_14s0108g00350 was lower at 30 °C than that at 40 °C. The dehydroquinate/shikimate dehydrogenase (SDH) members could affect the biosynthesis of SA and GA. Previous studies have shown that VvSDH3 and VvSDH4 are closely related to the synthesis of GA [28]. In this study, the transcription level of *VvSDH1* (VIT_05s0020g02030) was similar at the two dehydration temperatures. During the moderate dehydration stage, the expression level of *VvSDH2* (VIT_14s0030g00670) in berries at 40 °C was higher than that in berries at 30 °C. The expression level of *VvSDH3* (VIT_14s0030g00660) at 40 °C was higher than that at 30 °C only in the moderate dehydration stage, while the expression of *VvSDH4* (VIT_14s0030g00650) was higher than that at 30 °C at all dehydration stages. RT-qPCR analysis verified this result (Figure 8B). The above results showed that the dehydration process can upregulate the expression of GA biosynthesis genes in grape berries, especially at 40 °C, the temperature at which the transcription level for gallic acid biosynthesis was the highest.

NADP^+^/NADPH is an important metabolite for plants to cope with oxidative stress. The conversion from 3-dehydroshikimate (3-DHS) to SA or GA was also NADPH/NADP^+^ dependent. SA or 3-DHS could be used as the substrate for GA biosynthesis, and NADP^+^ as the cofactor in the process. The accumulation of NADP^+^ was more conducive to the biosynthesis of GA. In this study, the NADP^+^ content and NADP^+^/NADPH ratio were higher in berries treated at 40 °C (Figure 8C). Based on the analysis described above, the elevated expression of *VvSDH3* and *VvSDH4* and the accumulation of NADP^+^ may promote the accumulation of GA in raisins, and 40 °C was determined to be more conducive to the biosynthesis of GA.

## 4. Discussion

Postharvest dehydration could induce drastic changes in the transcription profile of grape berries [11]. In this study, the scattered points of each sample were distributed in an orderly manner, and the differences in transcription among samples were clearly related to the water loss state of grape berries. Similar to previous studies [11,19], genes related to various metabolic processes, such as hormone metabolism, redox, and major CHO metabolism, in the berries were continuously upregulated or inhibited by the dehydration stage. The metabolic profiles in postharvest grape berries during the dehydration process may be affected by dehydration conditions and the water loss rate [11,29,30]. The pre-treatment of olive oil used in the production of raisins can affect the composition and content of such polyphenols as anthocyanins, flavonols, and flavonoids in grapes. Olive oil-processed grapes have a faster dehydration rate and a higher content of flavonoids [30]. There were differences in the quality of postharvest grape berries under slow and accelerated water loss conditions, and the latter dampened the accumulation of stilbenes and flavonoids [27]. In this study, the contents of primary and secondary metabolites in raisins produced under different temperatures were different, indicating that the dehydration temperature could also modify the metabolic profile of grape berries during dehydration [19].

Postharvest temperature is closely related to the quality maintenance and development of postharvest grape berries [4], and the berry withering kinetic rates increase with increasing temperature [31]. Temperature could regulate the quality development of postharvest grape berries by affecting apoptosis-related enzyme activity and gene expression, and cell apoptosis was slower under low temperature conditions [32]. Moderate heat treatment has also been used to extend the shelf life of postharvest fruits and vegetables. Hot water treatment (HWT) can help reduce the postharvest loss and softening rate of fruits [33]. Moderate heat treatment could maintain the sensory quality of cucumbers [34] reduce freezing damage in kiwifruit [35], and change the expression of ethylene signal transduction genes to inhibit the post-ripening of tomatoes [36]. In contrast to the short-term high-temperature treatment, the treatment conditions in this study included continuous high temperature, and the berries changed more drastically at the level of transcription and metabolic profiles.

The water loss rate of grape berries can be adjusted by airflow. Previous studies have found that accelerating water loss by forced air accelerates the transcriptional programme of genes related to secondary metabolism and the evolution of metabolites, which is not conducive to the biosynthesis of stilbenes, and a slow rate of water loss is necessary for the accumulation of some metabolites [27]. In this study, the water loss rate of grape berries was adjusted by dehydration temperature, and the higher the temperature was, the faster the dehydration rate was. However, only 7654 genes showed the same transcriptional programme under the 40 °C and 30 °C dehydration modes, meaning that there was a difference between the effect of high-temperature water loss and forced-air water loss. Higher temperature (40 °C) not only accelerate the transcriptional programme of some genes but also induce new changes in the transcription of other genes.

Flavonoids are important nutrients in grape berries, are considered antioxidant molecules and free radical scavengers, and can locate and neutralize free radicals before they can damage plant cells [37]. High temperature and dehydration stresses affect the accumulation of flavonoids in grapes [13]. Short-term high-temperature treatment during the fruit development period affected the composition of tannins in Shiraz grapevine bunches and increased the proportion of galloylated skin flavan-3-ol subunits [38]. High night temperatures inhibited the accumulation of anthocyanins in grape berries, resulting in reduced fruit quality [16,39]. During postharvest withering, the content of anthocyanins, flavonols and other flavonoids in grape berries showed an overall downward trend [6]. Similar to previous studies, the content of various flavonoids in ‘Xiangfei’ berries also showed a decreasing trend during the process of dehydration, indicating that the high-temperature dehydration mode promotes the conversion or degradation of flavonoids. However, notably, the content of gallic acid and its derivatives accumulated during the process of grape dehydration [40]. Gallic acid was a product from a branch of the shikimic acid metabolic pathway [41]. 3-Dehyroshikimate is the branch point of the shikimate pathway, which can convert to SA by NADPH-dependent reduction under the catalysis of shikimate dehydrogenase (SDH) or GA by NADP^+^-dependent oxidation under the catalysis of SDH [28,42]. Habashi et al. [42] found that the expression of PgSDH1 was closely related to shikimate biosynthesis in pomegranate, while the increase in the expression levels of PgSDH3s and PgSDH4 led to the accumulation of GA and hydrolysable tannins under osmotic stress. Bontpart et al. [28] identified two shikimate dehydrogenases, VvSDH3 and VvSDH4, in grapes and showed that they were involved in gallic acid biosynthesis in grapevines with NADP^+^ as the cofactor through in vitro and transgenic tests. In this study, the expression levels of *VvSDH3* and *VvSDH4* in the berries treated at 40 °C were the highest, and the relative content of NADP^+^ was the largest, which may be the main reason that the highest content of gallic acid and its derivatives in the ‘Xiangfei’ raisins was observed at 40 °C. 

High temperature can cause changes in various metabolic pathways related to RNA, protein, cell development, hormone metabolism, primary metabolism and secondary metabolism in the fruit [19,43]. Many proteins, including heat shock proteins (HSPs), antioxidant enzymes, and those working in the electron transport chain of photosynthesis and glycolysis, may play key roles in grapevines when responding to and adapting to heat stress [44]. Heat shock proteins, such as HSP100, HSP70, and small heat shock proteins, are a class of molecular chaperones induced by heat stress in plants [43]; these proteins can prevent or reverse the inactivation and aggregation of heat-sensitive proteins and maintain or restore cell protein homeostasis [45,46]. sHSPs are considered to be the first line of defense under high-temperature stress [47]. Class I and II small heat shock proteins, as well as HSP101 in plants, protect protein translation factors during heat stress, and the ubiquitous sHSPs could act in vitro as molecular chaperones to prevent damage to heat-sensitive proteins [48]. Heat-shock proteins were also associated more closely with phosphorylation modifications of proteins under heat stress [49]. In this study, compared with the samples under 30 °C, the heat stress response-related genes of HSP or small HSP family in the grape berries under 40 °C were continuously activated in each stage of dehydration (Figure 7; Table S5), which indicated that the HSP members may play a key role in responding to heat stress in postharvest grape berries, and the induction of these genes was primarily temperature-dependent but not dehydration-dependent. Compared with other treatments under 30 °C and 40 °C, similar results were obtained at 50 °C at the mild water loss stage.

High-temperature stress destroys redox homeostasis in cells and causes peroxidation reactions [50,51]. The proteins related to the defense response and redox homeostasis in grapes were upregulated under high temperature, and they might function in the process of plant adaptation to high temperatures [33]. In this study, compared with samples at 30 °C, the expression of genes related to ascorbate and glutathione (VIT_07s0005g06290, VIT_08s0040g03150), dismutases and catalases (VIT_02s0025g04830), and thioredoxin (VIT_19s0014g01) in the redox pathway was continuously activated. The redox homeostasis pathway was also active in postharvest grape berries under HT stress. The hormone signalling pathway is also an important pathway in the response of plants to high-temperature stress [52]. Abscisic acid (ABA), jasmonic acid (JA), and salicylic acid (SA) together with ethylene could modulate plant defense responses to photooxidative and heat stress in sun-exposed fruit [53]. In this study, compared with the samples under 30 °C, some genes involved in abscisic acid, auxin, brassinosteroid, cytokinin, ethylene, and jasmonate synthesis and signal transduction process in the grape berries under 40 °C were activated. These hormone-signalling pathways may be coordinately involved in the response of postharvest grape berries to high temperature stress. In addition, a large number of transcription factors, such as AP2, MYB, and bHLH family members, were also activated under high-temperature conditions, and they may also participate in the high-temperature signal transduction process.

## 5. Conclusions

Postharvest dehydration temperature modulates the transcriptomic programme and flavonoid profile of grape berries. The high-temperature dehydration process can promote the accumulation of sugar, acid, and some phenols (mainly gallic acid and its derivatives) in the berries but inhibits the accumulation of proanthocyanidins, flavonols, and flavanols. Compared with 30 °C dehydration, 40 °C dehydration could accelerate the water loss rate and the transcription programme of 7654 genes and change the expression patterns of other genes. Meanwhile, high temperatures of 50 °C and 40 °C could induce continuous upregulation of genes related to the heat stress response and redox homeostasis in each stage of dehydration. The high-temperature dehydration process may promote the accumulation of gallic acid by increasing the expression levels of *VvSDH3* and *VvSDH4* and the production of NADP^+^. Considering the positive effects of flavonoids, proanthocyanidins and other phenols on human health, an appropriate dehydration temperature should be selected and applied when producing nutrient-rich raisins, and the temperature of 40 °C may be the optimal for phenolic components accumulation during the thermal dehydration of postharvest ‘Xiangfei’ grape berries.

## Data Availability

Grape berry transcriptome data are available in the National Center for Biotechnology Information (NCBI) database under the accession number SUB8954131.

## Supplementary Materials

The following are available online at https://www.mdpi.com/2304-8158/10/3/687/s1, Figure S1: Enriched pathways of differential metabolites in raisins produced at different dehydration temperature compared to the berry in the initial stage. Ratio represents the ratio of the number of differential metabolites annotated to the KEGG pathway to the total number of differential metabolites. Count represents the number of differential metabolites annotated to the KEGG pathway. The same below. Figure S2: Enriched pathways of differential metabolites among raisins produced at different dehydration temperature. Figure S3: The number of differential flavonoids, proanthocyanidins, and other phenols in different comparison groups. (A) The down- and up- regulated flavonoids, proanthocyanidins, and other phenols in different comparison groups. (B) The number of differential flavonoids, proanthocyanidins, and other phenols between raisins and grape berries. (C) The number of differential flavonoids, proanthocyanidins, and other phenols between raisins under different temperature. FC represent the fold change of differential components. VIP represent the p-value corrected by multiple hypothesis testing. The higher the VIP value, the higher the significance of difference. Figure S4: Differential flavonoids, proanthocyanidins, and other phenols in raisins produced at different dehydration temperature compared to the berry in the initial stage. Figure S5: The top 10 identified phenolic components with the most significant increase and decrease in different comparison groups. (A) The top differential components between raisins and grape berries. (B) The top differential components between raisins under different temperature. Figure S6: KEGG pathways enriched with downregulated DEGs between different groups. Table S1: Primers listed for RT-qPCR. Table S2: All metabolites identified in this experiment. All metabolites identified in the positive mode were listed in sheet 1, and identified in the negative mode were listed in sheet 2. Table S3: All flavonoids, proanthocyanidins, and other phenols identified in this experiment. Table S4: Overview of transcriptome data in this experiment. Table S5: Genes annotated by Mapman. The upregulated genes between different dehydration stage in samples treated at 30 °C and 40 °C were listed in sheet 1, the downregulated genes were listed in sheet 2, while the genes with similar transcription program between samples treated with 30 °C and 40 °C were listed in sheet 3.
