# Supplementary figures and images for: Postharvest Dehydration Temperature Modulates the Transcriptomic Programme and Flavonoid Profile of Grape Berries
Source: Foods. 2021 Mar 23;10(3):687. doi: 10.3390/foods10030687 (PMC8005005; doi:10.3390/foods10030687)

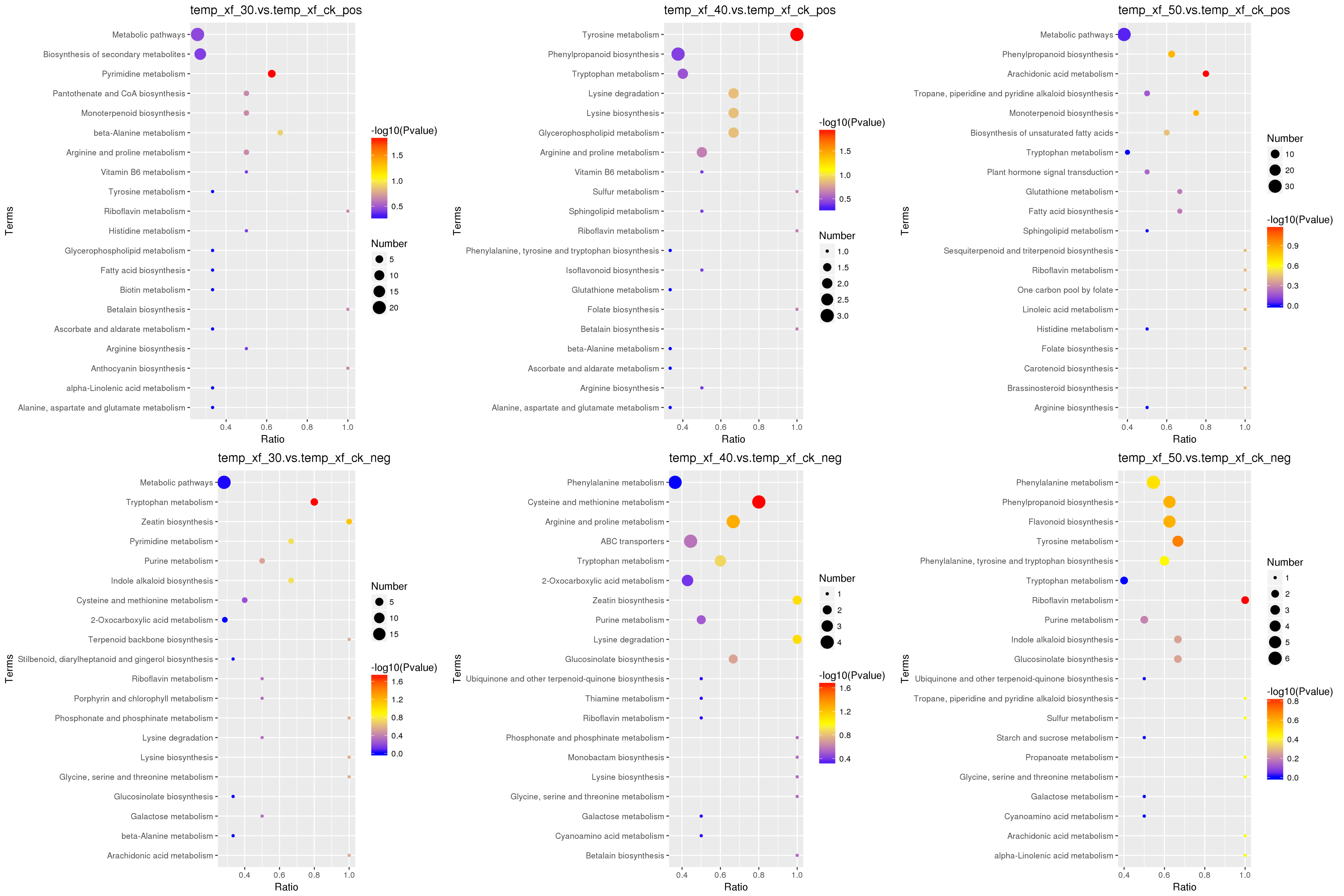

Supplement: Supplementary file 1 [file foods-10-00687-s001.zip › Fig. S1.png]

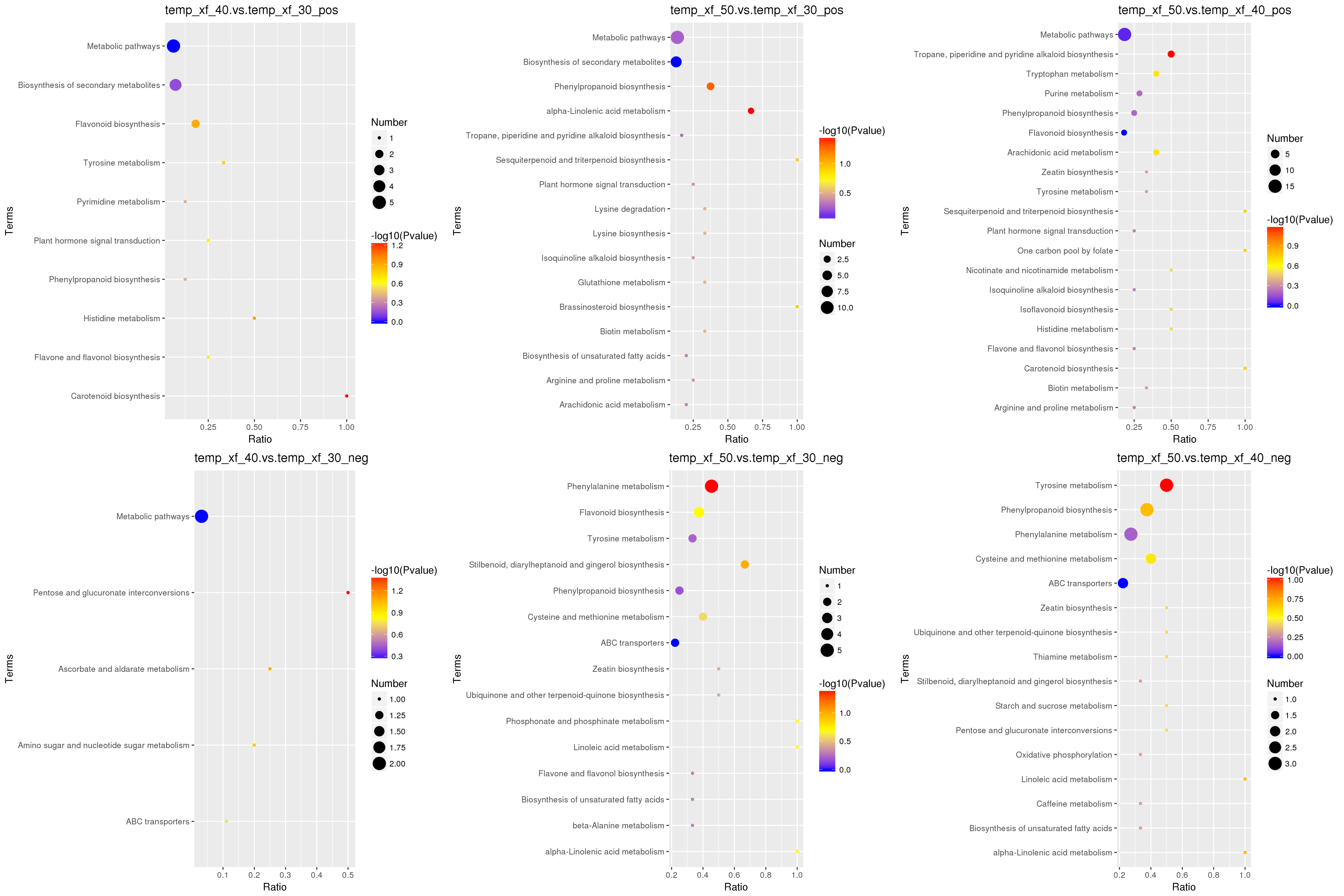

Supplement: Supplementary file 1 [file foods-10-00687-s001.zip › Fig. S2.png]

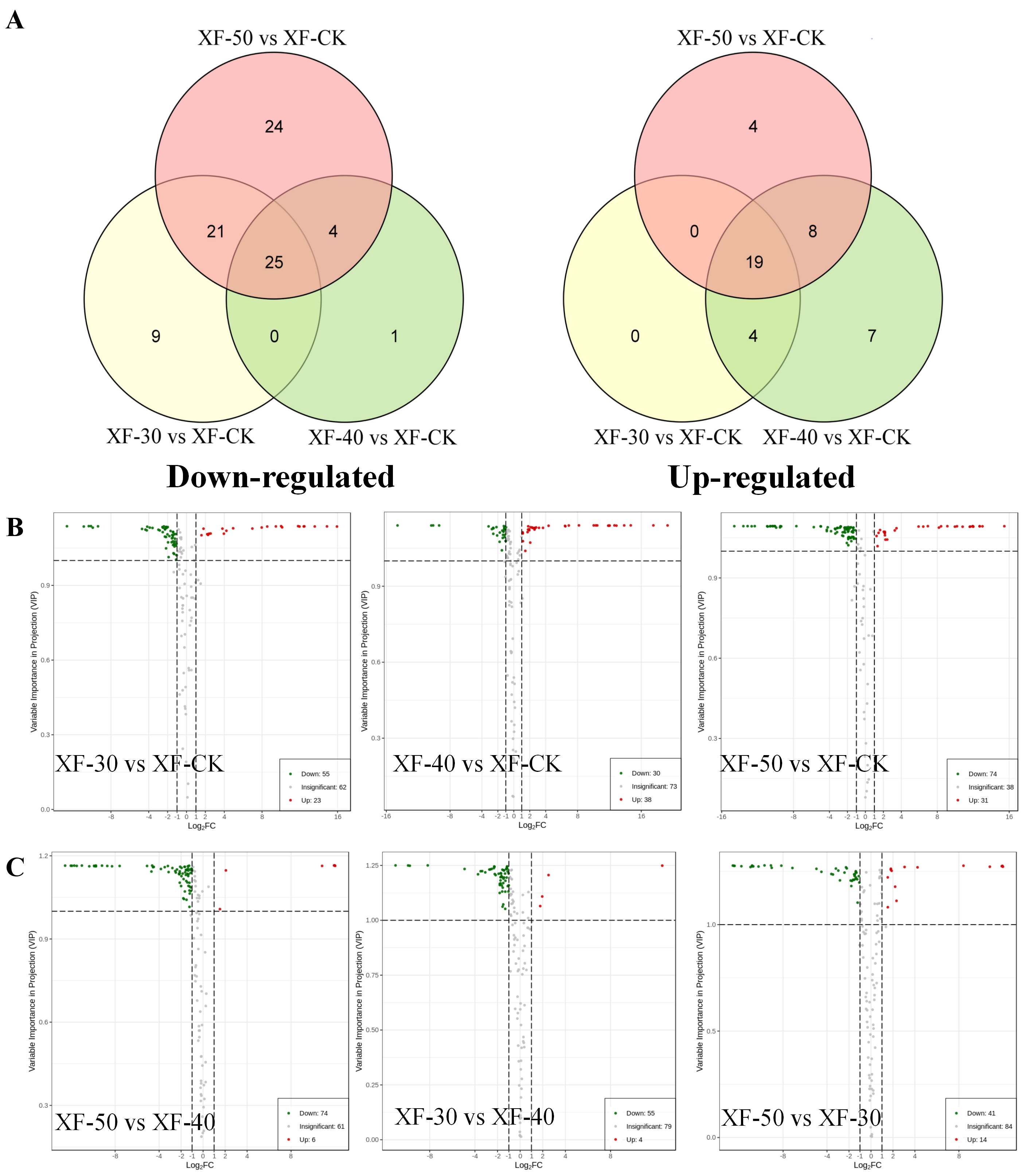

Supplement: Supplementary file 1 [file foods-10-00687-s001.zip › Fig. S3.png]

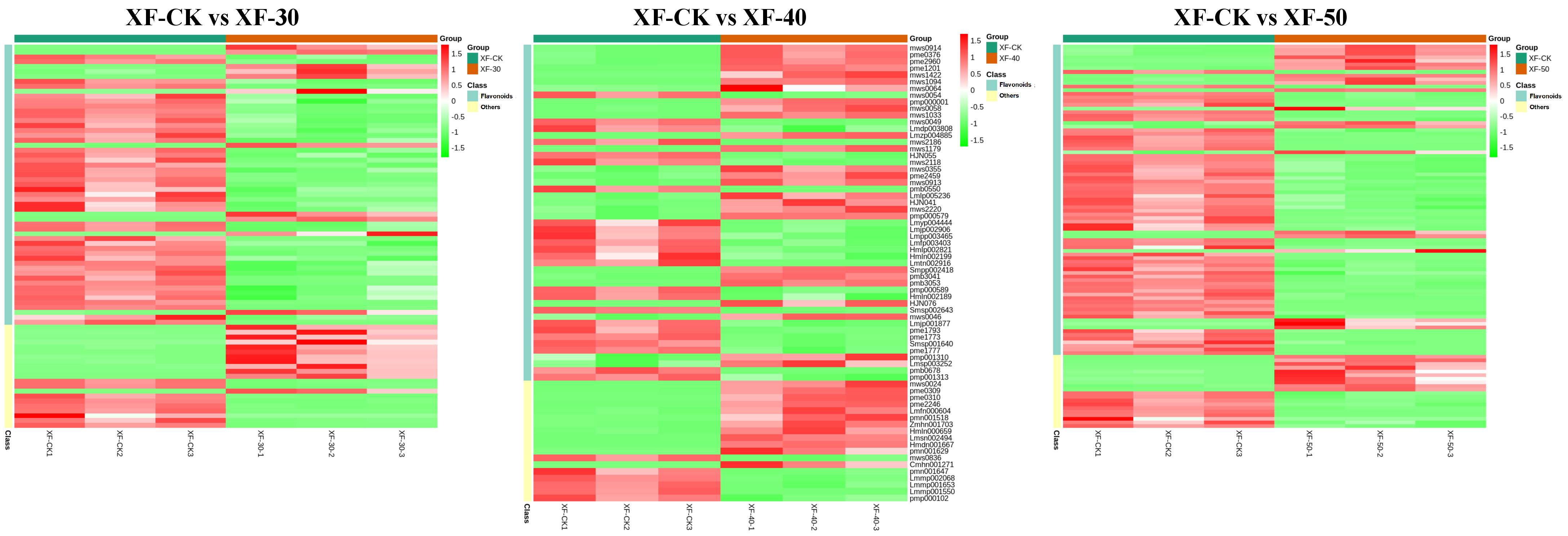

Supplement: Supplementary file 1 [file foods-10-00687-s001.zip › Fig. S4.png]

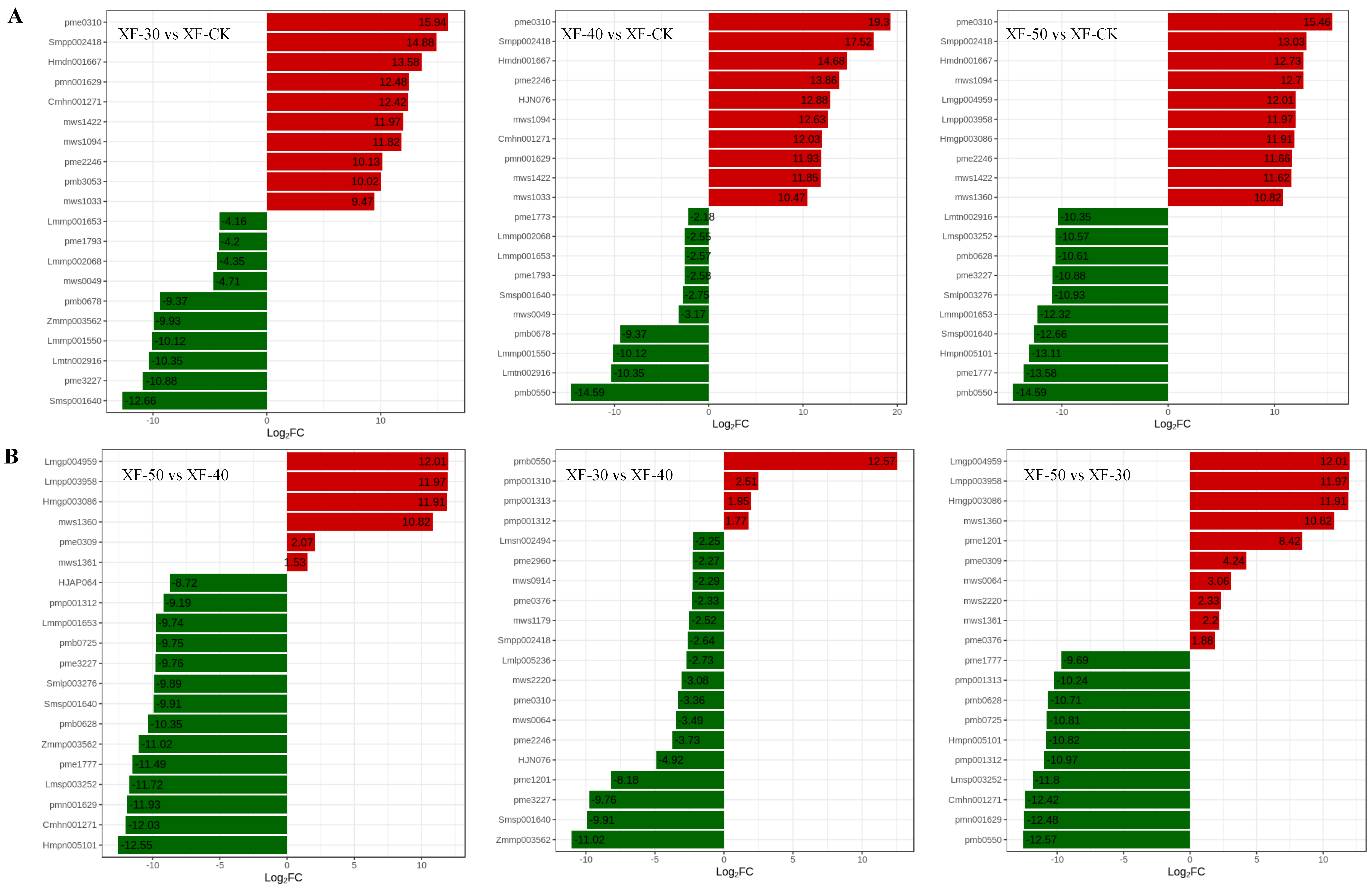

Supplement: Supplementary file 1 [file foods-10-00687-s001.zip › Fig. S5.png]

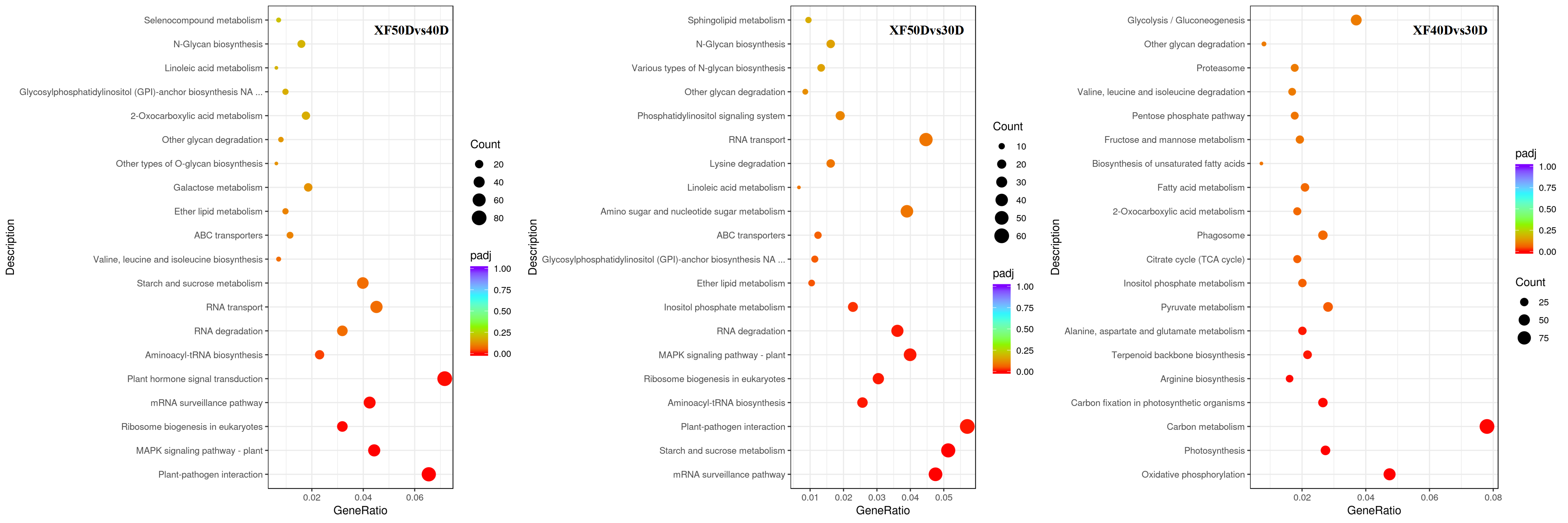

Supplement: Supplementary file 1 [file foods-10-00687-s001.zip › Fig. S6.png]
